# Apnea in a Two-Week-Old Infant Infected with SARS-CoV-2 and Influenza B

**DOI:** 10.1155/2022/2969561

**Published:** 2022-03-15

**Authors:** Radhika Maddali, Kelly L. Cervellione, Lily Q. Lew

**Affiliations:** ^1^Department of Pediatrics, Flushing Hospital Medical Center, Flushing, NY 11355, USA; ^2^Department of Clinical Research, Medisys Health Network, Jamaica, NY 11418, USA

## Abstract

Paucity of data exists on presenting symptoms and outcomes in infants with COVID-19. Reports of coinfection with COVID-19 and influenza B are sparse in the literature. Coinfection was uncovered during evaluation of neonatal apnea. Apnea has been reported in infants with SARS-CoV-2 infection, though it is rare. We describe a 2-week-old healthy term infant presenting with apnea and coinfection. The infant had a mild clinical course and complete recovery.

## 1. Introduction

Severe acute respiratory syndrome coronavirus 2 also known as SARS-CoV-2 causes coronavirus disease 2019 or COVID-19 [1]. Infected infants and children have a lower risk for symptomatic and severe infection than adults. Commonly reported clinical signs and symptoms of COVID-19 in infants include respiratory distress, cough, fever, nasal congestion, and poor feeding [2]. Since symptomatic COVID-19 in infants is not as common as in adults, presenting symptoms and disease course remain poorly characterized. Apnea has not been included as a symptom of COVID-19.

Infants who contract influenza are known to have a higher risk for severe illness than adults due to their immature immune system and smaller airway [3]. Infants combating coinfection may be at a risk for poorer outcomes than those with COVID-19 alone. Adults coinfected with COVID-19 and influenza [4–7] encountered higher morbidity and mortality than having COVID-19 alone [6–8]. We describe a 2-week-old healthy term infant presenting with apnea who was coinfected with SARS-CoV-2 and influenza B. The infant had a mild clinical course and complete recovery.

## 2. Case

A 2-week-old previously healthy girl presented with an apneic episode lasting three minutes while lying supine after a feed. The apneic episode was associated with stiffening of the body and turning red in color. There was no history of rhythmic or unusual facial movements. She experienced nasal congestion in the preceding two days that was not associated with fever, cough, or vomiting. She was born at term having a birth weight of 3365 grams (75^th^ percentile), length of 48.9 centimeters (50^th^ percentile), and head circumference of 33.5 centimeters (25^th^–50^th^ percentile). The mother tested COVID-19 negative at delivery. The patient's household consisted of two school-age siblings in addition to the parents. At admission, the infant's weight was 4082 grams (75^th^ percentile), temperature was 37.2°Celsius, pulse was 154 beats per minute, respiratory rate was 33 breaths per minute, and O_2_ saturation was 100% in room air. The heart and lung examinations were normal. There were no neurological deficits. Laboratory findings *included* a white blood cell count of 16.9 × 10^9^/L (16.9 K/uL), serum glucose of 6.27 mmol/L (113 mg/dL), calcium of 2.82 mmol/L (11.3 mg/dL), total carbon dioxide of 18 mmol/L, and C-reactive protein (CRP) of 0.00 nmol/L (<0.05 mg/dL). Urinalysis was *normal*. Chest radiograph revealed hyperinflated lungs with prominent central markings, no focal consolidation, no pleural abnormality, and unremarkable cardiothymic silhouette. SARS-CoV-2 PCR-NP (BioGX SARS-CoV-2 Reagents for BD MAX™ System) and influenza B viral antigens tested positive. A second SARS-CoV-2 PCR-NP was not obtained. Tests for influenza A virus, respiratory syncytial virus, and human metapneumovirus were negative. Electrocardiogram traced normal sinus rhythm without abnormal QT interval. Cranial imaging and cerebral spinal fluid analysis were not performed. She had an uneventful clinical course and was discharged after two days of observation.

## 3. Discussion

Children have fewer symptoms compared to adults [9] when coinfected with SARS-CoV-2 and influenza. Our patient experienced only nasal congestion in the two days preceding the apneic episode. She was feeding well and gaining weight. Domestic exposure to school-age siblings or by community spread was likely the mode of transmission. The siblings were asymptomatic and not tested. The mother tested COVID-19 negative at delivery, making vertical transmission unlikely. Our patient also did not have leukopenia or elevated CRP as previously reported in children infected with SARS-CoV-2 [10]. SARS-CoV-2 coinfection occurs at a higher rate with other respiratory pathogens [6]. Coinfection of SARS-CoV-2 with influenza A or B remains rare [8] and has been reported mainly in adults [11]. Influenza A is three times more common than influenza B and has been associated with apnea. Influenza B does not usually present with apnea. Coinfection with a second virus does not increase the frequency of apnea [12]. Similar to all respiratory viral illnesses, the most common symptoms of influenza B in children include fever, cough, and rhinorrhea. Those infected with influenza B tend to have milder symptoms and are symptomatic late into influenza season. Our patient presented at the beginning of influenza season and was too young to be protected by influenza vaccine. Whether one virus predisposed the infant to a second virus remains to be determined. The long-term effects of SARS-CoV-2 on young infants also remain to be determined. Our patient demonstrated full recovery without medical intervention.

Apnea occurs rarely in healthy full-term infants compared to preterm infants [13] with an incidence rate of one in 1000 [14]. Apnea presenting during the first weeks of life hints of “brief resolved unexplained event” (BRUE). In 2016, the American Academy of Pediatrics defined BRUE as an “acute event described by an observer with change in breathing, appearance, muscle tone and altered level of responsiveness lasting less than one minute in an infant younger than 12 months of age.” The term BRUE replaced “apparent life-threatening event” (ALTE) and “near-miss sudden infant death syndrome” (SIDS), terms previously used to designate apnea [15]. The history and physical examination of our patient met criteria for high-risk BRUE for age (<60 days) and duration of event (>one minute). Apnea associated with SARS-CoV-2 infection has been reported [13, 16, 17] and may be the presenting symptom [16, 17]. The COVID-19 pandemic and season for epidemic respiratory illnesses resulted in screening for viral respiratory illnesses. Tests for influenza A virus, respiratory syncytial virus, and human metapneumovirus, all known to be associated with apnea, were negative. BRUE was excluded when our patient tested positive for SARS-CoV-2 and influenza B.

## 4. Conclusion

We report the first case of apnea as the presenting symptom of SARS-CoV-2 and influenza B coinfection in a term infant. Apnea may be a symptom of COVID-19, of influenza B, or of coinfection. Healthcare providers should have a high index of suspicion for SARS-CoV-2 infection when infants have apnea and subtle respiratory symptoms. Based on our single case, coinfection does not necessarily predispose the infant to poor outcomes. Much larger studies are needed to characterize more accurately presenting symptoms and disease course in infants. It remains unclear from whom the infant contracted SARS-CoV-2 and influenza B.

## Data Availability

All data supporting this report are included in the report.
